# How social norms contribute to physical violence among ever-partnered women in Uganda: A qualitative study

**DOI:** 10.3389/fsoc.2022.867024

**Published:** 2022-09-01

**Authors:** Aloysious Nnyombi, Paul Bukuluki, Samuel Besigwa, Jane Ocaya-Irama, Charity Namara, Beniamino Cislaghi

**Affiliations:** ^1^Department of Social and Cultural Anthropology, University of Vienna, Vienna, Austria; ^2^School of Social Sciences, Makerere University, Kampala, Uganda; ^3^University of Stirling, Stirling, United Kingdom; ^4^Oxfam Novib in Uganda, Kampala, Uganda; ^5^Department of Global Health and Development, London School of Hygiene and Tropical Medicine, London, United Kingdom

**Keywords:** social norms, gender norms, violence against women and girls, physical violence, women's wellbeing

## Abstract

This paper contributes to the literature that studies how social norms sustain undesirable behavior. It establishes how norms contribute to intimate partner physical violence against women. First, norms organize physical violence as a domestic and private matter. Second, they organize physical violence as a constituent part of women's lives, thereby normalizing women's experience of abuse. Third, norms define appropriate boundaries within which male partners perpetrate violence. The findings draw essential information for social change interventions that target improvement in women's and girls' wellbeing. For social and behavioral programmes to change harmful norms, they have to deconstruct physical violence as a private matter, advance the de-normalization of physical violence, and dismantle acceptable boundaries within which violence happens.

## Introduction

Intimate Partner Violence (IPV) is a global public health and human rights concern. It is in the form of physical, sexual or emotional abuse. About 30% of women aged 15 and over experience physical and or sexual IPV during their lifetime. The prevalence is slightly higher in East Sub-Saharan Africa at 38.8% (Devries et al., 2013; Abramsky et al., 2018). In Uganda, the lifetime prevalence of physical or sexual IPV among ever-partnered women is 56%. Intimate partner physical violence among women stands at 45%, with slapping or having something thrown being the most frequent acts of physical abuse. Women that do not earn are more likely (20%) to report severe physical violence than those that earn (15%). Women who attended or attained primary education are more likely to be physically abused than those with university education (19% compared to 1%). Women with severe disabilities are more likely (61%) to have experienced physical violence than those without severe disabilities [43%] (Uganda Bureau of Statistics, 2021).

The Uganda government has made significant strides toward the protection of rights of women at risk or those experiencing IPV. The government has ratified and domesticated international and regional instruments through the Uganda Gender Policy (2007), The National Policy on Elimination of Gender-Based Violence in Uganda (2019), and the Uganda Domestic Violence Act (2010). The government has also implemented countrywide economic support programmes, namely the Women Entrepreneurship Programme, to address IPV risk factors. In addition, development partners are supporting efforts meant to address IPV. These are implementing safe and healthy relationship programmes for couples, family-based programmes, creating protective environments, strengthening economic support and providing survivor-centered services (Niolon et al., 2017). Recently, there has been growing interest in social norm-shifting programmes aimed at changing norms that drive IPV (Abramsky et al., 2018).

While social norms literature is multi-faceted (Legros and Cislaghi, 2019), much empirical research follows the theory advanced by Cialdini et al. (1990) that looks at social norms as people's beliefs about (1) what other people do (descriptive norms); and (2) what other people in the group approve of (injunctive norms). Norms are salient and often talked about—either praising those who conform or castigating those who do not. They help determine a collective understanding of acceptable attitudes and behaviors (Cislaghi and Heise, 2016). Moreover, norms can positively and negatively influence people's health: for example, push men to perpetrate IPV (because everyone does it) or protect them from perpetrating IPV (because friends would disapprove).

For social norms to exist, there must be a reference group of people whose behaviors and opinions matter to the person performing the behavior. These differ for the type of “behavior, situation and person considered” and for different norms (Bicchieri and Noah, 2017, p. 8). The reference group is located in physical proximity or distant from an individual (Alexander-Scott et al., 2016). These maintain norms through social approval or disapproval of one's actions, also called positive and negative sanctions (Cislaghi and Heise, 2016).

There is a burgeoning body of scholarship on norm-shifting interventions, for example, the SASA programme designed by Raising Voices and pilot tested in Kampala, Uganda. Such interventions are deliberate at catalyzing community-led change in norms and behaviors (Starmann et al., 2018). They cause social change through organized diffusion of positive reciprocal expectations. Programmes work with influencers who hold onto and practice positive behavioral expectations and have the skill and experience with the challenging process of exploring and questioning deeply held social beliefs. These persons support their networks to discuss negative reciprocal expectations around IPV and explore alternative behavioral expectations. As a result, there is the adoption of new social expectations on gender equality and violence, which brings about coordinated behavior change among several community members (Cislaghi et al., 2019).

Despite the expanding body of work, mainly on diagnosing, measuring and changing social norms (Cislaghi and Heise, 2016; Bicchieri, 2017; Legros and Cislaghi, 2019), there remains limited empirical evidence on how social norms shape abusive gender relations among intimate partners. This paper uses data from a study on ”social norms and violence against women and girls in Uganda” to address this gap. The theoretical argument that social norms distribute inequitable power relations, shape women and girl's unequal access to freedoms, build social acceptance of physical violence and entrench constructions of aggressive and dominant masculinities (Lundgren et al., 2018; Heise et al., 2019), present a critical framework that supports us in delineating essential pathways through which social norms contribute to physical violence among ever-partnered women.

## Methods

Data used to write this paper was collected under the study titled: “Formative Research on Social Norms and Violence against Women and Girls in Uganda.” The study sought to diagnose social norms that promote violence against women and girls and establish how these influence unequal gender relations. OXFAM NOVIB in Uganda and Uganda Women's Network (UWONET) under the ”ENOUGH: Together We Can End Violence against Women and Girls” campaign commissioned the study. Applied Research Bureau, a consultancy firm, designed and conducted the study. OXFAM and UWONET reviewed study documents and organized validation processes.

The study utilized a qualitative research design to diagnose existing social norms that influence physical, sexual, economic and bride price-related violence and collect nuances, specific contexts, complex relations and meanings assigned to these norms (Alexander-Scott et al., 2016; Cislaghi et al., 2016). This paper reports on norms that influence intimate partner physical violence.

Data was collected in the seven districts of Lira, Arua, Isingiro, Kabarole, Kamuli, Kotido, and Kampala. Regional representation criteria informed the final selection of study districts. Focus Group Discussions (FGDs) and In-depth Interviews (IDIs) were used to collect data on intimate partner physical violence. Each method targeted a separate group of ever-partnered adults (25 and above) and young (18–24 years) women and men. We conducted 14 sex-separate FGDs with adults and four sex-separate FGDs with young people in intimate relationships.

The FGDs included a vignette of a hypothetical scenario where the husband slaps and kicks the wife after confronting him about engaging in an extra-marital relationship. The intention was to stimulate participants thinking about physical violence's beliefs and expectations. The researchers developed the vignette and finalized it following pilot testing.

In addition, we conducted fourteen (14) IDIs with female survivors of IPV, including three living with a disability and 29 IDIs (16 women and 13 men) with local leaders, staff from non-government organizations, a Member of Parliament and the central government (see Table 1).

**Table 1:** Study participants.

	**Arua**		**Isingiro**		**Kabarole**		**Kamuli**		**Kotido**		**Kampala**		**Lira**	
	**Urban**	**Rural**	**Urban**	**Rural**	**Urban**	**Rural**	**Urban**	**Rural**	**Urban**	**Rural**	**Urban**	**Rural**	**Urban**	**Rural**
FGD with adults (25+ years)	1 female	1 male	1 male	1 female	1 female	1 male	1 male	1 female	1 female	1 male	1 male	1 female	1 female	1 male
FGD with young people (18–24 years)					1 female			1 male	1 female					1 male
IDIs with survivors	1	1	1	1	1	1	1	1	1	1	1	1	1	1

### Data analysis

The data analysis process commenced during data collection when the researchers noted ideas and issues emerging from the interviews. After data collection, we familiarized ourselves with the data regarding its depth and breadth. This process started with verbatim transcription of the audio-recorded data. After data transcription, we repeatedly read the data, actively searching for meanings, patterns and themes to prepare for coding. We identified the codes and then matched them with data extracts demonstrating that code. After coding and collating all the data, the focus moved beyond codes to themes. We sorted the different codes into potential themes and collated all the relevant coded data extracts within the identified themes. Subsequently, we described and provided explanatory accounts of the coded data.

### Ethical considerations

We conducted the study according to the ethical and safety recommendations for intervention research on violence against women (WHO, 2016). Makerere School of Social Sciences Ethics Committee and the Uganda National Council of Science and Technology approved the study protocol, tools and procedures. All researchers were trained in research ethics and passed the Research, Ethics and Compliance curriculum. Also, we trained researchers on how to safely refer women requesting assistance to available local services and sources of support. Participation was voluntary, and ongoing informed consent was obtained from all study participants to ensure ongoing, voluntary participation and continued safety. Participants were discouraged from sharing intimate personal details about their experiences with violence; anyone who wanted to discuss such experiences was given a list of local GBV services and offered the opportunity to speak with someone immediately. We protected the participants' confidentiality and privacy by anonymising the data. We did not use the participants' names and limited information about the participants' location.

## Results

We present results as follows: (1) descriptive norms (what most people in a group do) that influence physical intimate partner physical violence (2) injunctive norms (what other people in the group approve of) that influence physical intimate partner physical violence. To understand mechanisms through which norms contribute to intimate partner physical violence, we report on nuances, specific contexts and meanings assigned to each norm identified.

### Descriptive norms

#### Husbands beat their wives under given circumstances

One of the descriptive norms identified is that “husbands beat their wives under given circumstances.” Men sanction this norm when women fail or delay fulfilling their ascribed gender roles. Discussions with some older men show that the intention is to discipline or instill discipline. They argued that this form of discipline is within acceptable limits and expected to achieve a given outcome—a woman recognizing her mistake and meeting the different gender expectations. They added that this differentiates what they engage in from the mainstream acts of physical abuse.

What happens in a home is controlled. We discipline our wives. We do not beat to hurt but to discipline. We have to punish them so that they get to the correct path. When she bleeds or breaks her hand, that is not disciplining; you have now started to hurt the mother of your children (Male adult, Kamuli district).

The decision to ”discipline” is often not one-off. It instead happens after a series of demands for one to change. It could also be from the continued commentary from the husband's close network on the spouse's behavior that contrasts the set gendered expectations. The husband then decides to “discipline” the woman for bringing shame to him.

It is never an easy decision. Your friends could keep talking about your wife and how she behaves like a young girl who does not know what is expected of her. So it reaches a stage when you can longer freely interact with your friends. So it is wise to beat her so that she changes (Male adult, Kampala district).

The act of “disciplining” is expected to happen within the homestead, even when transgression of gendered expectations occurs in a public space. It is expected that the husband expresses his dissatisfaction with the wife's behavior in their bedroom and punishes her in the same space. Discussions with older women revealed that the bedroom is the ideal place for this to happen.

Often we do not stay alone as husband and wife; you have relatives that come to stay, you have your children, and your parents may stay with you. So, when my husband beats me in our bedroom, I will be very okay. No one will get to know what happened; they continue perceiving me in the same way as they did before, not as someone who is beaten or wrongs her husband (Female adult, Kamuli district).

Some ever-partnered men in rural and urban locations believed that wife-beating is shameful. They argued that men that do it lose respect among their friends, family and community. Some male perpetrators deny engaging in the act or provide justifications for their actions, especially if the spouse's family intervenes. He could blame the woman for engaging in immoral behavior (e.g., extra-marital affairs, theft) to gain sympathy.

Some young and older men in urban areas opined that there is no ground on which wife-beating is acceptable. They did acknowledge that this could happen however advised that men should desist from engaging in such acts. Some noted that there are alternatives to wife-beating that men can engage in that do not cause physical harm.

It is not a good thing at all, and there is no reason one should give for engaging in it. Therefore, I advise my fellow men not to beat their wives (Male adult, Kampala).

Some ever-partnered women and men believed that wife-beating is a physical gesture that shows that the husband loves and cares for the wife. They added that husbands that choose to beat their wives are interested in maintaining the relationship. These point out the spouse's wrongdoings and punish them. The intention is to discourage further engagement in similar behavior.

If a loved child did something terrible, wouldn't you punish her? You do it because you love her; that is the same thing we do when our lovely wives do something wrong. We do it because we love them (Male adult, Isingiro district).

I know they are laughing, but we all know that when a man does not beat you, you start questioning whether he is interested in you, you do something wrong, but he keeps quiet. So it could be that person does not love you, and he is planning to leave you soon (Female adult, Arua district).

#### No one should intervene when a husband beats his wife

The other descriptive norm that influences physical intimate partner violence is “no one should intervene when a husband beats the wife.” Discussions with ever partnered women and men reveal that wife-beating is a private matter (between husband and wife) that does not call for the intervention of the “outsiders.” When a woman perceives her experience as violent and unfair, the expectation is that she seeks an audience with the spouse to talk about and resolve the matter at hand. She does not have to ask for the intervention of the “outsiders,” and neither are “outsiders” expected to intervene in a “small” matter, as one of the adult male participants referred to wife-beating, “a rather small matter that does not require another person's intervention.”

When individuals or groups intervene in the matter, husbands blame spouses for inviting “outsiders,” even when they are unaware of the individuals or groups' intention to intervene. Survivors of physical abuse reported that this results in repeated and more violent perpetration of abuse.

Whenever the chairpersons (local leaders) would come to talk about what was happening to me, that man would complain that they wanted to destroy our relationship. He would also blame me, that I was the one who asked the chairman to confront him, yet I was not the one. And then he would beat me, much more than he ever did (Survivor of physical violence, Kabarole district).

However, interviews with women and men show that elders from the man and woman's families can intervene when the physical violence persists. Nonetheless, their role is limited to the reconciling of the two parties.

When a man beats you today, the other day and the other week, your family can call on his family, and they talk to the man to see that this does not happen again (Adult woman, Lira district).

Discussions with women and men revealed that law enforcement officers often intervene in such matters when notified. It is common for men to provide an audience to the officers out of the fear of imprisonment. However, often their role is limited to reconciliation. The spouse must pay the medical bill when the woman requires medical attention. That said, some officers do not intervene. They argue that the case does not have legal implications, and the husband and wife can reach a peaceful agreement.

If it were between you and your husband, the officer would say, “you go back to your husband's home; this is something that you must address with your husband” (Adult woman, Isingiro district).

#### Men that pay the bride price own and control their spouses

The other descriptive norm reported is that “men that pay the bride price own and control their spouses.” Discussion with adult and young women and men show that payment of bride price symbolizes a transfer of rights and responsibilities, mainly control and ownership, from the woman's family to the spouse. They explained that control and ownership involve ensuring her wellbeing, approving decisions, and disciplining her when she transgresses gender expectations.

Women belong to the man. Therefore, when a woman's family receives the bride-wealth, the woman becomes his property (Interview with a cultural leader, Arua district).

Women who decide to leave the relationship have to return bride-wealth. In the districts of Isingiro, Kabarole and Kotido, older women reported that this influences women's decision to stay in abusive relationships. Often, the family is unwilling to return the items; in other instances, they are not in a position to purchase similar items.

You get to think of the things they brought, cattle, and our fathers cannot allow us to give them back, so you stay with that man (Adult woman, Kotido district).

In our culture, someone brings cows, not 1 or 2 but about 5. So when you want to separate from the man, he will ask you for his cows. Truthfully where you will get them (Adult women, Isingiro district).

However, interviews with adult men in urban centres revealed that not all men expect the woman's family to return bride-wealth. They rather see separation as an opportunity for a fresh beginning.

I was fed up with her. The moment she left, I was relieved. Some asked that I ask for my cows, but I no longer cared about that (Adult male, Isingiro district).

#### Experience of physical violence is a domestic matter that should not be shared

The other descriptive norm identified was “experience of physical violence is a domestic matter that should not be shared.” Ever partnered women and men reported that such experiences are not shared since they bring shame to the family. Some participants recited proverbs that expound on why such experiences should not be shared to emphasize the importance of upholding this norm.

Family matters are usually considered private. They always say, “A woman should not tell the whole world what they are going through” (Survivor of Violence, Kampala district).

Nonetheless, some women transcend this behavioral expectation as they share their experiences with persons they can confide in. Others report their experience to law enforcement officers. Notably, the persons with whom the experience is shared often reiterate that this is a domestic matter that should not have been shared in the first place and asks the woman to handle the issue with the spouse.

I did talk to a few people in our community about my marriage issues, that is, my mother and some other old lady who happened to be my neighbor. However, she told me I should not discuss such matters with anyone. So I did cry and suffered in silence (Survivor of Violence, Arua district).

Some persons that survivors confide in blame them for their situation, especially when the cause of the abuse was the failure to complete gender roles. Some, especially family members, could choose to visit the husband and apologize on behalf of the family for the wife's inability to perform her role.

If you are beaten for being “big-headed,” they will send you back to your marital home, claiming that you were in the wrong. They will even come home and apologize to the man (Adult woman, Kampala district).

Participants noted that husbands that get to find out that the spouse talked to someone physically assault their partners. The intention is to force her into keeping silent about her experience.

Whenever my neighbor talked to my husband about what I had told her, he would become more aggressive. He would severely beat me up. I then decided to keep quiet; I could not tell anyone (Survivor of Violence, Arua district).

Discussions with adult men show that husbands who choose to share their experience with perpetrating abuse draw sympathy from some community members. They think that men never disclose what is happening in their lives and only do so in extreme cases. In addition, adult women noted that men that disclose such experiences blame their spouses for pushing them into perpetrating abuse.

However, adult women and men noted that there are circumstances where it is acceptable for women to share their experiences. For example, when physical abuse persists or when this results in life-threatening harm. That said, they can only share their experience with elders from the woman's and man's families.

### Injunctive norms

### A wife should tolerate physical violence perpetrated by the husband

One of the injunctive norms reported is “a wife should tolerate physical violence perpetrated by the husband.” Ever partnered adult women noted that tolerance is a comment on how well a paternal aunt prepared the woman for marriage. Paternal aunts encourage women to be strong and brave as they experience physical abuse. As a result, women who endure abuse can raise their children with their partners and preserve family honor.

Interviews with survivors of physical abuse revealed that women who share their experiences are made aware of the behavioral expectation of being tolerant. Also, they are comforted that the spouse will change his ways soon, providing a sense of hope that builds resilience to the abusive experience.

I talked to my mother about the situation I was going through. She advised me to stay in the marriage and care for the children. I also talked to the chairman (local leader), and he said the same, stay in the marriage. I was abused, I was tortured, but I was strong (Survivor of violence, Kamuli district).

Discussions with adult women show that some women eventually leave abusive relationships, although blamed and ridiculed for their decision. Those that leave their children behind are labeled irresponsible.

I did leave that relationship; I could not tolerate it anymore. The problem was that I left my children with the man. My mother kept on blaming me for that. She told me that that man would marry soon and the woman would not treat the children well (Survivor of violence, Kampala district).

Interviews with adult men and women revealed that some male parents of abused women warmly welcome them back to their households and stand by them when other family members criticize or blame them for what happened.

Truthfully my father was happy that I finally decided to come home. One day he found my brother blaming me for what happened, and he asked him never to think of me that way (Survivor of violence, Kampala district).

When I learned my child was mistreated, I asked her to return home. She took some time to decide to, but she finally did (Adult male, Lira district).

### Women have to behave in a way that upholds men's domination

Another injunctive norm identified was that “women have to behave in a way that upholds men's domination.” Interviews with adult women and men show that women should be respectful and obedient toward their husbands. They should manifest this in their daily interactions. Disrespectful and disobedient women are punished by beating or being asked to return to their parents' homes.

When I ask her, she responds arrogantly, and you know what comes next: a beating. Then, finally, you slap her once or twice because of disrespect (Adult male, Kamuli district).

### Men and women perform different roles

Another injunctive norm identified is that “men and women perform different roles”—communities ridicule and name-call women and men that fail to complete their roles. Notably, husbands beat up wives, complaining about their failure to meet their roles. Also, wives that fail to complete their ascribed roles are punished, sometimes through beating.

Discussions show that there are changes in the manifestation of this norm. For example, women's entry into the job market has relegated some roles to house-helps. Nonetheless, they have retained the supervision of these roles.

Many families now have maids, so the maids deal with things like preparing warm water, but still, it is my responsibility to see that she boils the water; if she does not, my husband shall blame me, not her (Adult woman, Kampala district).

### Reporting physical abuse to the police casts an evil spell on the family

Another injunctive norm identified is that “reporting physical abuse to police casts an evil spell onto the family.” Adult women mainly described the evil spell in terms of the hard times that the children and their mother would endure when the breadwinner was in prison.

She told me that if I reported my husband and he got imprisoned, I would have brought a curse on my kids and family, and she advised me not to do it. She asked, who would provide for the children? (Survivor of violence, Kampala district).

Discussions with adult women revealed that women that report physical abuse experience discrimination from their peers and families. When they seek support, peers and family members remind them that they had the breadwinner arrested. Again, they are told that the only support that can be provided is for children; this could only be gotten if they are out of her care.

I did report him to the police, and he was imprisoned. It reached a time when I needed help, but whomever I reached out to, refused to offer support. Instead, they told me I was to blame for what was happening (Survivor of violence, Isingiro district).

Discussions with law enforcement officers revealed that some women choose to withdraw the case when they realize they shall lose out on their source of support. As a result, women keep in abusive relationships and harden men perpetrating abuse, given that they cannot be held accountable for their actions.

## Discussion

We found evidence of descriptive and injunctive social norms that sustain physical violence against women in intimate relationships. The findings build on earlier evidence, in low-and-middle-income countries, on behaviors typical or approved of in contexts of physical violence (Allen and Raghallaigh, 2013; Carlie and Trott, 2017).

The social norms identified have both a direct and indirect relation to the practice of physical violence. A direct relation between norm and the practice happens “in situations where a norm and behavior are matched” (Cislaghi and Heise, 2018, p. 6). The relationship is indirect when the norm does not explicitly relate to the practice but contributes to the shared behavioral expectation of perpetrating physical violence. Examples of such norms drawn from this study include men and women performing different roles; men who pay the bride price, own and control their spouses; and reporting physical abuse to the police casts an evil spell on the family.

Findings show that social norms build a local discourse on physical violence against women in intimate relationships. The discourse organizes physical violence as a domestic and private matter, which contrasts with the feminist discourse that argues for attending to violence against women as a public concern (MacKinnon, 2006; Krizsán et al., 2007). The norms construct physical violence as a private concern attended to by intimate partners or, in exceptional circumstances, a matter to be handled by the family. The implication is that norms define persons and institutions where matters of physical violence are reported and resolved, which places state institutions and actors at the margins as communities do not recognize their role in addressing women's experiences of violence. When women reach out to the state actors, they advise them to return to their private spaces. The decision is embedded in social norms and not the existing legal options provided by the Uganda Domestic Violence Act (2010).

These realities present evidence of some mechanisms through which a culture of silence on women's experiences is built or strengthened. Treating violence as a domestic and private matter pushes discussions on the perpetration of physical violence out of the public realm (McAlister et al., 2021). The informal rules and threats of punishment that the norms present force women to keep their experiences to themselves. The culture of silence also extends to groups within the community that norms do not recognize as competent in intervening in cases of physical violence. Findings suggest that the behavioral rules that maintain a culture of silence are primarily enacted when a man's gender capital (dignity and respect in the community) are threatened.

The local discourse also organizes physical violence as a constituent of women's life worlds. It constructs violence as an everyday practice (Tolman et al., 2003; Messerschmidt, 2012)—-“husbands beat their wives under given circumstances,” “a wife should tolerate physical violence perpetrated by the husband.” In this context, physical violence means love and care, which presents moral justifications for men's perpetration of violence, which then constructs violence as a practice to achieve a greater good within society. Consequently, there is men's limited accountability for their violent behaviors. Women and girls are responsible for ensuring that men do not victimize them. Those victimized are blamed for their limited attention to the various informal rules that the social norms present.

The local discourse also defines appropriate boundaries for physical violence and associated practices. For example, although generally, physical violence is treated as a private matter, the informal rules allow one to report a case of violence to state authorities once her experience goes beyond what is considered appropriate. In addition, even though the family is one of the spaces where cases of violence are reported, the informal rules limit their role to reconciling the two parties and restoring the gender order.

We found evidence that women (and men) think of contesting particular social norms. However, the norms rule out specific pathways for which this person can opt (Chambers, 2005). For example, women often think of reporting the spouse to law enforcement agencies, but they choose not to because norms describe the act as one that brings about an evil spell. Some women exercise their agency to challenge the existing norms. However, this causes a change in the manifestation of the norm, not the meaning attached to the social norm. For example, women join the labor market. Nonetheless, retain the responsibility of supervising housework done by the house help.

## Conclusions

As an increasing body of work in LMIC focuses on improving social and behavioral change programming, there have been advances in the understanding of what works in supporting communities to achieve change in harmful norms, especially those affecting women's and girls' wellbeing. As such, there is a good understanding of practical strategies that social norm change interventions must adopt to achieve normative changes. To further strengthen the effectiveness of these strategies, we see it essential to understand the different ways social norms contribute to negative behaviors affecting women and girls. This offers programmers practical action areas to be attentive to if they are to cause normative change. We identify three pathways through which social norms contribute to intimate partner physical violence: (i) norms organize physical violence as a domestic and private matter, (ii) norms organize physical violence as a constituent part of women's lives, and (iii) norms define appropriate boundaries within which male partners perpetrate violence. We believe that social norm-shifting interventions that deconstruct physical violence as a private matter, advance the de-normalization of physical violence and dismantle acceptable boundaries within which violence happens can cause tremendous achievements in addressing physical violence among ever-partnered women.

## Data availability statement

The original contributions presented in the study are included in the article/supplementary material, further inquiries can be directed to the corresponding author/s.

## Ethics statement

The study protocol was reviewed and approved by Makerere School of Social Sciences Ethics Committee and the Uganda National Council of Science and Technology. Written informed consent to participate in this study was provided by the participants.

## Author contributions

All authors listed have made a substantial, direct, and intellectual contribution to the work and approved it for publication.

## Funding

This work was funded from Oxfam Novib in Uganda.

## Conflict of interest

The authors declare that the research was conducted in the absence of any commercial or financial relationships that could be construed as a potential conflict of interest.

## Publisher's note

All claims expressed in this article are solely those of the authors and do not necessarily represent those of their affiliated organizations, or those of the publisher, the editors and the reviewers. Any product that may be evaluated in this article, or claim that may be made by its manufacturer, is not guaranteed or endorsed by the publisher.
